# Laparoscopic surgery for rectal cancer, specimen extraction: transanal or transabdominal?

**DOI:** 10.1186/s12893-023-02059-7

**Published:** 2023-06-16

**Authors:** Fatemeh Shahabi, Ala Orafaie, Majid Ansari, Zahra Gholami Moallem, Ali Mehri, Maryam Hejri Moghadam, Reza Roshanravan, Abbas Abdollahi, Mahboobeh Rasouli

**Affiliations:** 1 Endoscopic and Minimally Invasive Surgery research center, Mashhad University of Medical Sciences, Mashhad, Iran; 2 Ghaem Hospital, Mashhad University of Medical Sciences, Mashhad, Iran; 3 Department of Biostatistics, School of Public Health, Iran University of Medical Sciences, Tehran, Iran

**Keywords:** Rectal cancer, Laparoscopy, Specimen extraction, Transanal, Transabdominal

## Abstract

**Background:**

Comparison of natural orifice specimen extraction (NOSE) and transabdominal specimen extraction (TASE) in colorectal surgery remains controversial. Herein, we aimed to perform a retrospective analysis on surgical outcomes of NOSE and TASE at three hospitals in east of Iran.

**Method:**

Consecutive locally advanced rectal adenocarcinoma patients who underwent laparoscopic surgery using either NOSE or TASE from 2011 to 2017 were recruited. These patients were followed-up till 2020. Data, including postoperative complications, long-term overall and recurrence-free survival were analyzed retrospectively.

**Results:**

239 eligible patients were included in this study. 169 (70.71%) patients underwent NOSE, and 70 (29.29%) patients underwent TASE. Although this study has achieved similar outcomes in terms of overall and recurrence-free survival, metastasis, circumferential margin involvement as well as complications of intra-operative bleeding, obstruction, anastomosis-fail, rectovaginal-fistula in women and pelvic collection/abscess in both groups, we observed higher rates of locoregional recurrence, incontinency, stenosis and the close distal margins involvement in NOSE group and also obstructed defecation syndrome in TASE cases.

**Conclusion:**

According to our findings, NOSE laparoscopic surgery showed significantly higher incontinency, impotency, stenosis and involvement of the close distal margins rates. Nevertheless, considering the similarity of long-term overall and recurrence-free survival, metastasis, circumferential margin involvement, NOSE procedure is still could be considered as a second choice for lower rectal adenocarcinoma patients.

## Introduction

Colorectal cancer is the third most common cancer and the second leading cause of cancer death worldwide. One-third of colorectal cancer cases are rectal cancer [1]. The incidence of rectal cancer is higher in Asia in comparison with western countries [2]. Technically, rectal cancer surgery remains one of the most demanding procedures as the quality of the dissection may influence oncological outcomes [3–7].

In early 20th century when the laparoscopy technique was introduced, a revolution was happened in surgery. Although the use of laparoscopy has been increasing in colorectal surgery, the focus has now shifted to further refinement of this technique [8]. Despite the advantages of laparoscopy, the complications such as surgical site infections and incisional hernias have been reported [9]. To mitigate such problems, natural orifice specimen extraction (NOSE) and transabdominal specimen extraction (TASE) have been developed for rectal cancer surgery. Reduction of the number and size of abdominal incisions with a possible decrease in postoperative pain, earlier gastrointestinal function, and decrease in hospital stay duration were the advantages of NOSE over TASE in conventional laparoscopy in benign colorectal disease. However, there is a lack of conclusive evidence on its benefits in the field of rectal cancer surgery. Implantation of tumor at the specimen extraction site and also pelvic sterility during surgery are two major concerns in NOSE technique [10, 11]. Hence, which technique is the better approach for rectal cancer remains controversial.

This study has aimed to retrospectively analyzed and compared the postoperative complications, cancer recurrence and long-term survival of the laparoscopic NOSE and TASE in rectal adenocarcinoma patients in east of Iran.

## Methods

### Study design, patients and variables

**Fig. 1:**
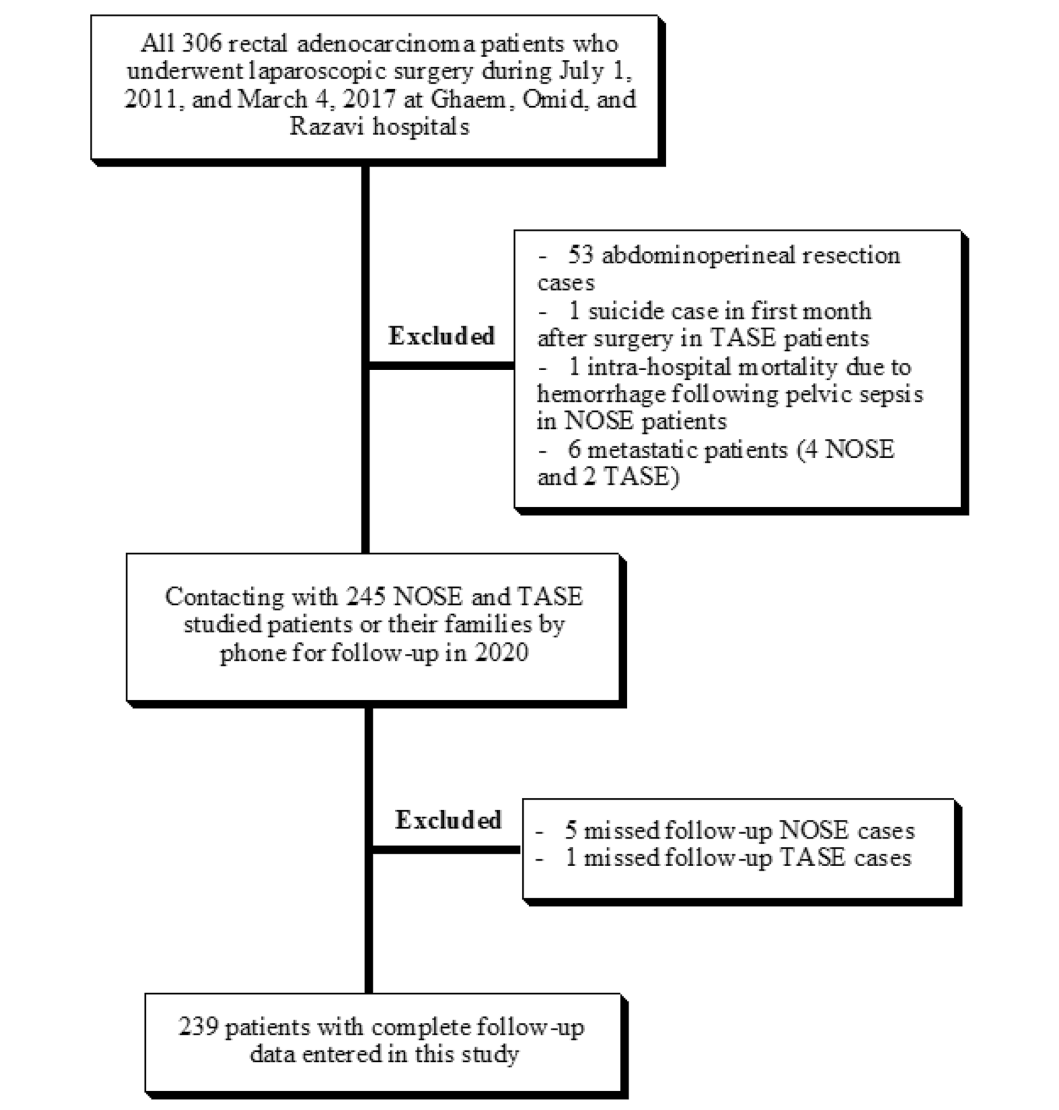
Diagram of patient’s recruitment in this study

A retrospective cohort of 300 non-metastatic patients with locally advanced rectal adenocarcinoma diagnosed in Mashhad, Iran was evaluated. The diagram of patients’ recruitment in the study is shown in Fig. 1. The studied patients underwent laparoscopic surgery in tow surgical procedure types: Transanal and Transabdominal. Before the operation, 98.7% of these patients have received neoadjuvant chemoradiation therapy, and at least a month after chemoradiation, the surgery was performed. The patients were administered Capecitabine 500 twice a day as part of their chemotherapy treatment, and underwent a total of 28 sessions of radiation therapy, with a total radiation dose of 5400 rad. 75.3% of the patients have received adjuvant chemotherapy after surgery. All patients involved in the study had a score of II or III in the ASA indexing score. The ASA score is a classification system used to evaluate a patient’s physical status before surgery. It ranges from 1 to 6, with higher scores indicating a greater risk of complications. Studied patients were followed-up based on standard rectal cancer surveillance till fifth year after curative surgery. After this time periods, phone calls have been made to patients annually. Demographic and clinical variables of age at diagnosis, gender, surgical procedure techniques, operation time, tumor location (from the anal verge, three parts are defined as follows: the lower rectum, 0 to 5 cm; the middle rectum, 6 to 10 cm; and the upper rectum, 11 to 15 cm), recurrence type, metastasis pattern, circumferential resection margins (CRM) involvement, distal resection margins (DRM) involvement, and postoperative TNM stage of the patients were examined in this study. In addition, intra-operative complications of bleeding and postoperative complications of obstruction, anastomosis-fail, impotency in men, rectovaginal-fistula in women, pelvic collection/abscess, incontinency based on the cleveland clinic incontinence score and stenosis were compared. A part of the data of this study was collected from the colorectal cancer registry (No: 4,001,728), Mashhad University of Medical Sciences, Mashhad, Iran.

### Surgery method

In modified lithotomy position with laparoscopic approach, after complete abdominal exploration, the inferior mesenteric artery was exposed and high ligated and then ligation of inferior mesenteric vein was done at the inferior border of the pancreas just lateral to the duodenum. Complete mobilization of the splenic flexure colon, left colon, sigmoid and rectum, were done. The procedure was completed in two different techniques in this stage. In the first group (NOSE), circular incision was done just above dentate line in rectum and enough distal to the inferior border of rectal tumor and after complete dissection, wound protector was inserted and the rectum, sigmoid and left colon were pulled through the anus and resection was done at enough proximal margin and the specimen was removed. An anastomosis (with stapler or hand sewn techniques) was created between the left colon and distal of the rectum or anal canal. In second group (TASE) after complete mobilization, rectum was cut at enough distal margin with stapler and then specimen removal was done through the wound protector from abdomen with Pfannenstiel incision and resection was done at enough proximal margin and the specimen was removed. The anastomosis created between left colon and rectum with circular stapler in this technique.

### Statistical analysis

The continuous and categorical variables were reported in the mean ± standard deviation (SD) and frequency (percentage), respectively. After checking relevant assumptions, the Chi-squared test or Fisher’s exact test was performed to compare the categorical variables and to compare continuous variables, after checking the normality, independent two sample t test or Maan-Whitney test which one applicable was used. Recurrence/metastasis-free survival (RMFS) was calculated from the date of primary surgical procedure to the date of diagnosis of the first locoregional recurrence or metastasis disease or the last follow-up in patients without recurrence and overall survival (OS) was calculated from the date of primary surgical procedure to the date of death or last follow-up. The Kaplan-Meier (KM) curves of all patients for all two survival outcomes adjusted for surgical procedures were presented, and the log-rank test was used to compare the differences between the survival curves. The three and five-year RMFS and OS rates of patients at each level of surgical procedure variables were calculated and to compare these rates, Chi-squared goodness of fit test was used. In order to investigate the effect of clinical and demographic variables on the binary outcome, binary logistic regression was utilized. Significant variables at α = 0.2 in univariate were candidate to enter the multivariable model. The analysis was performed using SPSS version 26.0 (Chicago, IL, USA). The significance level was considered 0.05.

## Results

**Fig. 2:**
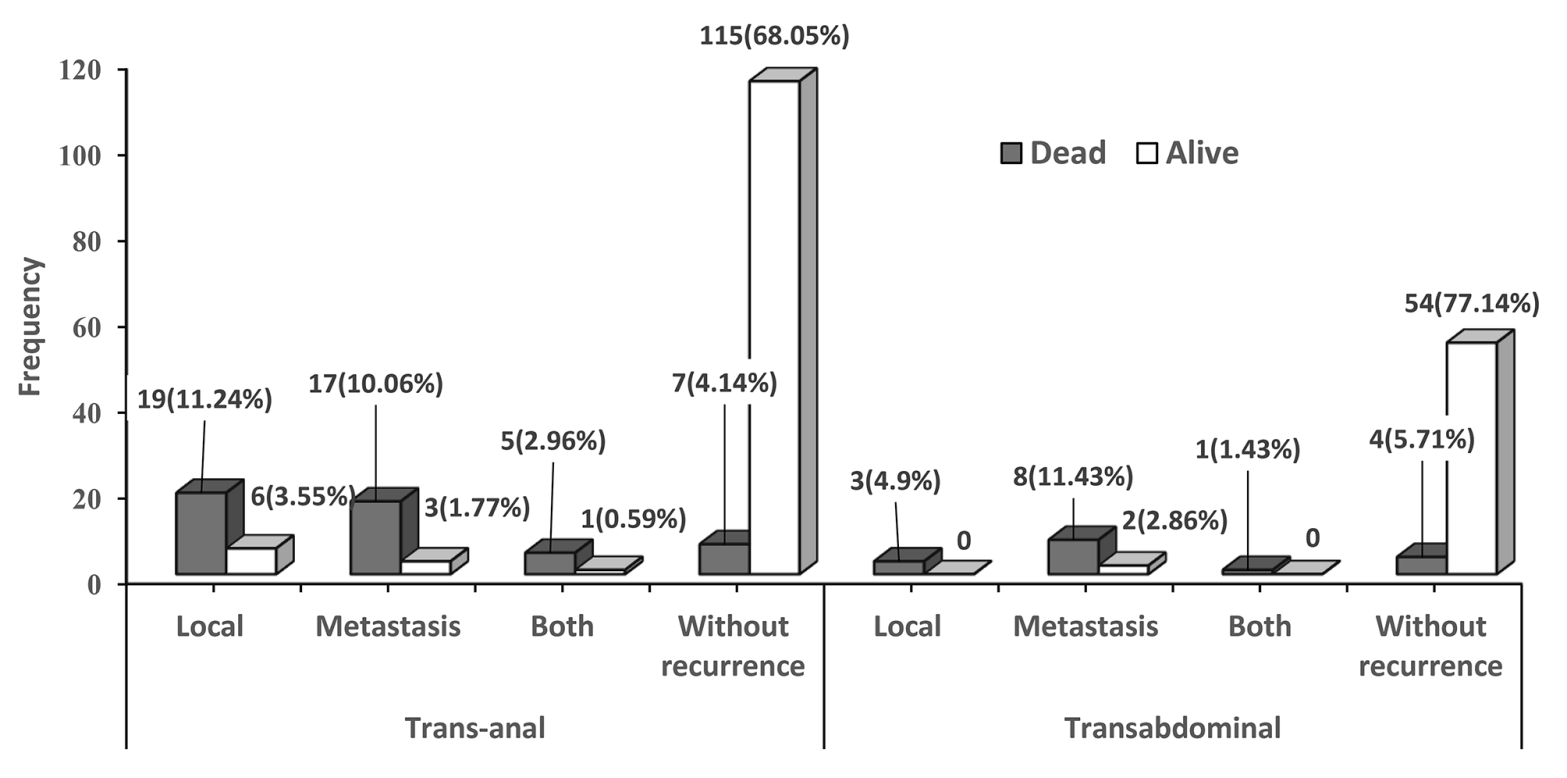
Frequency distribution of mortality and recurrences status according to surgical techniques

239 patients were included in this study. There were 138 (57.74%) males and 101 (42.26%) females, and the median (IQR = interquartile range) age of the patients was 53(16). 169 (70.71%) patients underwent NOSE, and 70 (29.29%) patients underwent TASE. The median (IQR) follow-up time (survival time) for all the patients was 65(37) months; about NOSE and TASE groups were 72 (39), and 53(24) months, respectively. Seventy (29.29%) patients (54 (31.95%) in NOSE and 16 (22.86%) in TASE) died during the study. In addition, 30.18% of NOSE patients and 20% of TASE patients experienced at least one type of recurrence. Frequency distribution of mortality and recurrences according to surgical techniques is provided in Fig. 2. Comparison of demographic and clinical characteristics between NOSE and TASE surgical procedure group presented in Table 1. The frequency of distribution of the patients were homogeneous between the surgical groups in terms of the gender and disease stage (P > 0.05). However, the mean ± SD age of patients in the TASE group was significantly higher than that of the NOSE group (P = 0.002). It is clear that the mean time of NOSE surgery is longer than TASE (P < 0.001). Locoregional recurrence was significantly higher in NOSE patients than in the TASE group (P = 0.022), however, a significant percentage of patients in the NOSE group had lower rectal tumors (P < 0.001). In term of metastasis pattern there was no difference between both groups (P = 0.120).

**Table 1:** Comparison of demographic and clinical characteristics between NOSE and TASE surgical procedure group

Characteristics		NOSE (n = 196)	TASE (n = 70)	*P*
**Age at diagnosis**, (mean ± SD, year)		51.60 ± 12.42	57.10 ± 11.50	0.002*
**Operation time**, (mean ± SD, min)		206.90 ± 18.34	195.37 ± 16.28	< 0.001*
**Lymph nodes extraction**, mean ± SD		4.80 ± 4.93	5.60 ± 5.30	0.362
**Positive lymph nodes**, mean ± SD		0.97 ± 2.29	0.54 ± 1.07	0.826
**Gender, N (%)**	Male	103(60.95)	35(50)	0.119
	Female	66(39.05)	35(50)	
**Postoperative TNM stage, N (%)†**	pCR	51(30.18)	21(30)	0.662
	T1,2	41(24.26)	13(18.57)	
	T3,4	31(18.34)	17(24.29)	
	N positive	44(26.03)	19(24.14)	
**Tumor location, N (%)**	Low	123(72.78)	16(22.86)	< 0.001*
	Mid	32(18.93)	29(41.43)	
	Upper	14(8.28)	25(35.71)	
**Local recurrence, N(%)**		25(14.79)	3(4.29)	0.022 *
**Distant metastasis, N(%)**		20(11.83)	10(14.29)	0.603
**Both recurrence (local + distant metastasis), N(%)**		6(3.55)	1(1.43)	0.677
**Metastasis pattern, N(%)**	Liver	6(23.08)	8(72.73)	0.120
	lung	9(34.61)	1(9.09)	
	Bone	2(7.69)	0	
	Brain	1(3.85)	1(9.09)	
	Multi-site	4(1.54)	0	
	Other and unknown	4(1.54)	1(9.09)	
**Circumferential resection margin†,N (%)**	Free	164(97.04)	70(100)	1.000
	Close margin	2(1.18)	0	
	Involved	1(0.59)	0	
**Distal resection margin†, N (%)**	Free	153(90.53)	68(97.14)	0.023*
	Close margin	12(7.10)	0	
	Involved	2(1.18)	2(2.86)	

pCR = pathological complete response

†missing for 2 patients

*significance at α = 0.05

In order to investigate the effect of demographic and clinical variables on the locoregional recurrence, regression analysis was performed. As shown in Table 2, in the presence of age, tumor location, surgical technique and distal resection margin variables, the odds of locoregional recurrence increased by 5.41% in N positive TNM staging patients than pathologic complete response patients.

**Table 2:** The effect of demographic and clinical baseline variables on locoregional recurrence using binary logistic regression model in studied patients

		Univariate analysis			Multivariable analysis		
Variables		Odds ratio	S.E.	P	Odds ratio	S.E.	*P*
Age		0.98	0.02	**0.136**	0.98	0.02	0.386
Gender	Male	-	-	-			
	Female	0.87	0.41	0.735			
TNM stage	pCR	-	-	**-**	-	-	-
	T1,2	3.43	0.72	**0.085**	3.56	0.73	0.081
	T3,4	3.29	0.73	**0.105**	3.45	0.77	0.107
	N positive	5.41	0.67	**0.012**	5.41	0.69	0.014 *
Surgical technique	NOSE	-	-	-	-	-	-
	TASE	0.26	0.63	**0.031**	0.27	0.70	0.063
Tumor location	low	-	-	**-**	-	-	**-**
	Mid	0.42	0.57	**0.126**	0.59	0.61	0.388
	Upper	0.68	0.58	0.680	1.31	0.70	0.704
Circumferential margin involvement	Free	-	-	-			
	Closed/involved	3.83	1.24	0.279			
Distal margin involvement	Free	-	-	-	-	-	**-**
	Closed/involved	2.74	0.62	**0.103**	1.62	0.65	0.462

*Significant at α = 0.05

pCR; pathologic complete response

Stoma statuses of these patients were summarized in Table 3. Permanent stoma condition was reported in 26.03% of NOSE and 10% of TASE patients. However, 73.37% of NOSE patients had not stoma. Although, some free-stoma patients suffered from incontinency (incontinency reported in Table 4), they were managed by pelvic floor physiotherapy, medication and appendicostomy.

**Table 3:** Stoma status of the studied patients

Status	NOSE, N(%)	TASE, N(%)	*P*
**Surgery without primary stoma**	49(28.99)	7(10)	< 0.001 *
**Surgery + stoma and then closure**	64(37.87)	55(78.57)	
**Late stoma and then closure**	11(6.51)	0	
**Late permanent stoma**	19(11.24)	0	
**Primary stoma closed and then permanent stoma**	11(6.51)	0	
**Still stoma**	14(8.28)	7(10)	
**Unknown**	1(0.59)	1(1.43)	

* Significant at α = 0.05

**Table 4:** Comparison of intra-operative and postoperative complications between NOSE and TASE surgical procedure groups

Characteristics		NOSE	TASE	*P*
**Intra-operative bleeding, N (%)**		5(2.96)	1(1.43)	0.491
**Intra-operative internal organ injury, N(%)**		1(0.59)	1(1.43)	0.501
**Obstruction, (%)**		16(9.47)	4(5.71)	0.340
**Anastomosis fail, N (%)**		24(14.20)	5(7.14)	0.128
**Pelvic collection/abscess, N (%)**		13(7.69)	4(5.71)	0.588
**Ventral hernia, N(%)**		5(2.96)	3(4.28)	0.604
**Rectovaginal fistula in females, N (%)**		2(3.03)	0	0.543
**Obstructed defecation syndrome, N (%)**		3(1.77)	8(11.43)	0.003 *
**Frequency of defecation, N(%)**		12(7.10)	6(8.57)	0.695
**Urinary disorders, N(%)**		9(5.32)	6(8.57)	0.383
**Fistula, N(%)**		6(3.55)	0	0.184
**Stenosis, N (%)**		38(22.5)	1(1.4)	< 0.001 *
**Impotency in males, N (%)**		44(42.72)	8(22.86)	0.013 *
**Incontinency in patients without stoma in 6 months after surgery†, N(%)**	None	35(20.71)	36(51.43)	< 0.001 *
	1–5	37(21.89)	14(20)	
	6–10	40(23.67)	8(11.43)	
	11–15	29(17.69)	4(5.72)	
	16–20	13(7.69)	0	

*Significant at α = 0.05 †still stoma patients were removed for evaluation of incontinency

The patients were compared with respect of the surgical complications’ frequency between the two groups in Table 4. The frequency of different degrees of incontinence complications had not the same distribution in both surgical groups. A significant percentage of NOSE patients suffered from various degrees of incontinence, and this percentage was significantly higher than the TASE group (P < 0.001).

**Fig. 3:**
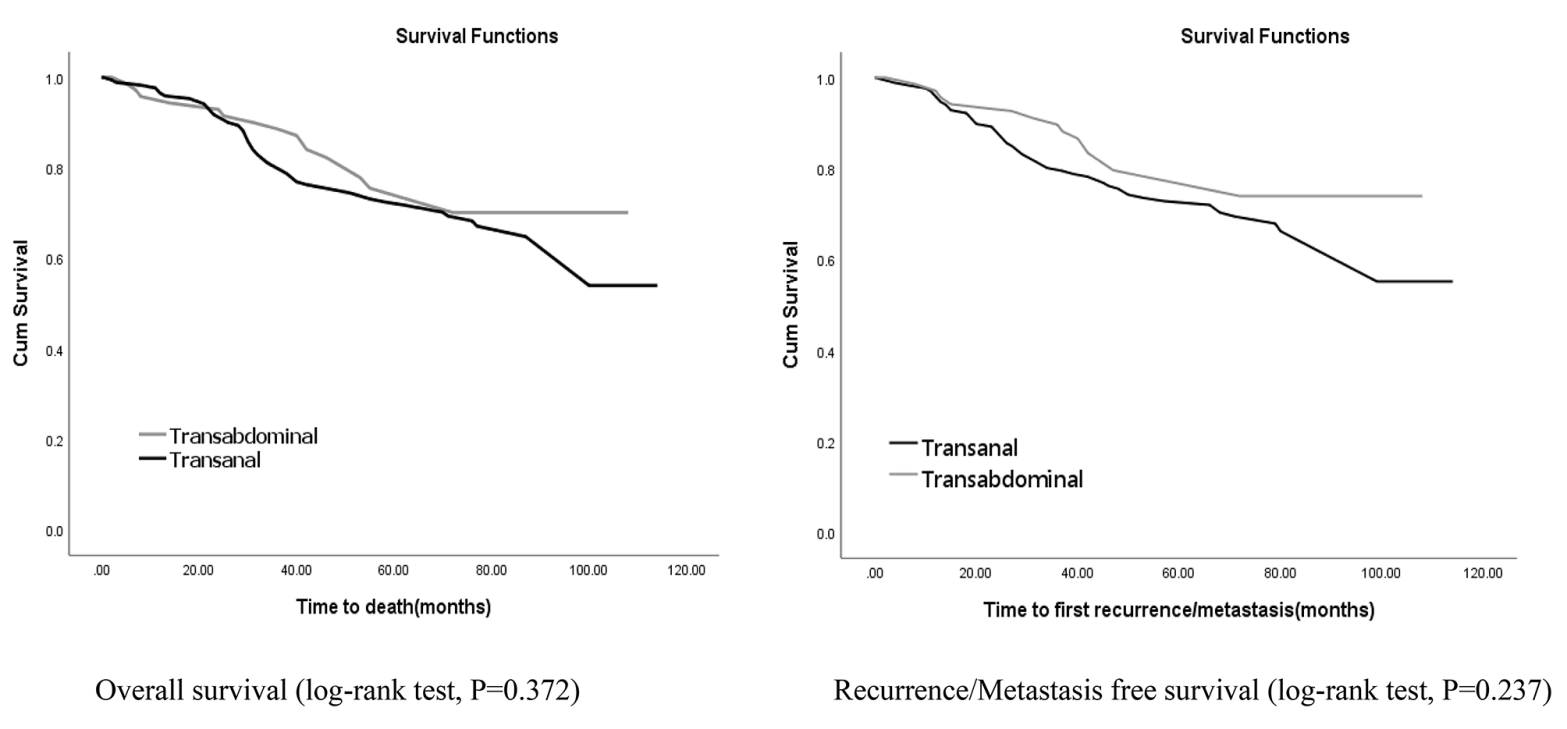
Kaplan-Meier survival plot of patients in this study

The Kaplan-Meier (KM) OS and RMFS curves for all rectal cancer patients included in this study were demonstrated in Fig. 3. As illustrated in this Figure, no statistically significant difference observed between the survival curves of the two surgical procedures. The log-rank test also confirmed this result. The three-year OS rates of 80% (83,77) and 90% (94,86) belongs to NOSE and TASE patients (P = 0.443), respectively. Moreover, the five-year OS rates (95% CI) for NOSE and TASE were 72% (76,68) and 72% (78,66), respectively. The results were close for RMFS (95% CI) rates. The three-years rates were not statistically different for NOSE vs. TASE [(P = 0.400), (80% (83,77) vs. 91% (94,88)]. In addition, five-years rates were as follows 72% (76,68) vs. 76% (82,70) (P = 0.742).

Severity of postoperative complications of our patients based on received treatments was reported in Table 5 with Clavien-Dindo classification [12]. The results of the chi-square test showed that patients in the NOSE group experienced complications with a higher grade. This has shown that the frequency of complications which required intervention (under general anesthesia or without anesthesia) is higher in NOSE group.

**Table 5:** The comparison of postoperative surgical complications based on Clavien-Dindo classification system

			NOSE	TASE	*P*
**Grade I**			176(62.19%)	60(85.71%)	0.001 *
**Grade II**			0	0	
**Grade III**	**Grade IIIa**		43(15.19%)	3(4.29%)	
	**Grade IIIb**		63(22.26%)	7(10)	
**Grade IV**	**Grade IVa**		0	0	
	**Grade IVb**		0	0	
**Grade V**†			1(0.35%)	0	

Significant at α = 0.05

Intra-operative bleeding(n = 6), internal organ injury(n = 2) and anastomosis fail(n = 10) during surgery were omitted from this classifications/ † this case was excluded from this study but died in the hospital due to sepsis after surgery, it was added to this classification

## Discussion

Rectal cancer is a global health concern, as its incidence is increasing in younger populations. Fortunately, during the past 40 years the treatment options for rectal cancer have expanded, leading to better outcomes and improved quality of life [13]. Laparoscopy has been associated with a clear progression in the management of rectal cancer patients and has shown notable improvement in postoperative measures, such as pain, first bowel movements, and hospital stays [14]. Over the past two decades, refinement of laparoscopy has led to the development of NOSE. However, its safety and oncological benefits compared with TASE in laparoscopic surgery remains controversial [15, 16].

Wang et al. reported that the operative time was longer in NOSE surgery compared to TASE [17]. In the present study as well, the operative time was longer in the NOSE group. Many factors might affect the operative time, such as complexity of surgery, experience of surgeon and patient’s BMI, etc. The longer time for the procedure of NOSE might be because of another reason. In this technique, surgeon needs to put the patient in two different positions which may take time and make this operation longer than TASE.

In terms of locoregional recurrence, there was a significant increase after NOSE surgery compared to TASE, while the other studies had reported no significant difference between two groups [17–20]. Although the frequency of locoregional recurrence was significantly higher in NOSE patients, multiple regression analysis indicated that postoperative TNM stage was the only significant effective variable on locoregional recurrence and in the presence of the other variables, surgical techniques had no statistically significant effects.

Recently, it has been shown that there is no significant difference between NOSE and TASE groups in terms of proximal and distal resection margins involvement [19]. However, our NOSE group had significantly higher close distal resection margin involvement.

Several studies have indicated that there is no significant difference in disease-free survival and overall survival at 3 years and 5 years between two groups [17–20]. We also did not encounter any difference in overall survival and recurrence/metastasis free survival in both groups. The comparable long-term curative effect of NOSE and TASE, suggest that both surgeries can be safe for use in rectal cancer treatment.

In the present study, it was found that except for incontinency, stenosis, impotency in men and also obstructed defecation syndrome, other postoperative complications were comparable in both techniques. NOSE surgery group showed higher rates of incontinency, stenosis, impotency in men, while TASE group indicated higher rates of obstructed defecation syndrome. Some free-stoma patients in both groups suffered from incontinency. To deal with this problem, they were managed by pelvic floor physiotherapy, medication and appendicostomy. In the other studies, NOSE and TASE were comparable in terms of overall postoperative complications [17–21].

The mentioned surgical studies are threatened by case selection biases. This subject is a limitation of our study. The patients were selected based on surgeon preference and patient characteristics including, age, tumor location and cosmetic subject. In order to deal with this problem, the effects of variables such as age, tumor location, involvement of margins, etc. were investigated on the occurrence of locoregional recurrence in a regression analysis. Locoregional recurrence had a significant difference between the two groups.

TASE has disadvantages such as the requirement of a 5–7 cm incision for specimen removal which typically leads to a risk of adhesions and incision hernia [22–24]. On the other hand, NOSE laparoscopic surgery is mini-invasive and has shown better cosmetic results, while in our study this procedure showed higher rates of postoperative complications. However, considering the similarity of these two procedures in terms of survival rate and metastasis, it seems that NOSE might be a second choice for patients with low rectal cancer. Moreover, it has been reported that non-obese patients and also females are more suitable for NOSE surgery [21].

## Conclusion

In conclusion, both procedures can be effective methods for rectal cancer specimen extraction. Although NOSE causes more postoperative complications including incontinency, impotency and stenosis, this method is comparable with TASE in terms of metastasis rate, three- and five-years OS and RMFS rates. On the other hand, abdominal incision is an important disadvantage of TASE procedure. Therefore, it is better to evaluate each patient individually. Hence, we suggest that, the characteristics of both the specimen and the patient should be considered for making a final decision.

## Data Availability

The datasets used and/or analysed during the current study are available from the corresponding author on reasonable request.

## Abbreviations

NOSE: natural orifice specimen extraction
TASE: transabdominal specimen extraction
CRM: circumferential resection margins
DRM: distal resection margins
SD: standard deviation
KM: Kaplan-Meier
OS: Overall survival
RMFS: Recurrence/metastasis -free survival
